# Effects of Silicon vs. Hydroxytyrosol-Enriched Restructured Pork on Liver Oxidation Status of Aged Rats Fed High-Saturated/High-Cholesterol Diets

**DOI:** 10.1371/journal.pone.0147469

**Published:** 2016-01-25

**Authors:** Jorge A. Santos-López, Alba Garcimartín, Pinar Merino, M. Elvira López-Oliva, Sara Bastida, Juana Benedí, Francisco J. Sánchez-Muniz

**Affiliations:** 1 Department of Pharmacology, School of Pharmacy, Universidad Complutense de Madrid, Madrid, Spain; 2 Department of Nutrition and Food Science, School of Pharmacy, Universidad Complutense de Madrid, Madrid, Spain; 3 Departament of Biomedical Sciences, Teaching Unit of Toxicology, School of Pharmacy, Universidad de Alcalá, Madrid, Spain; 4 Departament of Physiology, School of Pharmacy, Universidad Complutense de Madrid, Madrid, Spain; Universitat de Lleida-IRBLLEIDA, SPAIN

## Abstract

**Background:**

Pork is an essential component of the diet that has been linked with major degenerative diseases and development of non-alcoholic steatohepatitis (NASH). Previous studies have. Previous studies have demonstrated the in vitro antioxidant activity of silicon (Si). Furthermore, when Si is added to restructured pork (RP) strongly counterbalances the negative effect of high-cholesterol-ingestion, acting as an active hypocholesterolemic and hypolipemic dietary ingredient in aged rats.

**Objective:**

This study was designed to evaluate the effects of Si vs hydroxytyrosol (HxT) RP on liver antioxidant defense in aged rats fed cholesterol-enriched high saturated/high cholesterol diets as a NASH model.

**Methods:**

Four diets were prepared: Control RP diet (C) with non-added cholesterol; Cholesterol-enriched high-saturated/high-cholesterol control RP diet (CHOL-C) with added cholesterol and cholic acid; Si- or HxT-RP cholesterol-enriched high-saturated/high-cholesterol diets (CHOL-Si and CHOL-HxT). Groups of six male Wistar rats (1-yr old) were fed these modified diets for eight weeks. Total cholesterol, hepatosomatic index, liver *Nrf2* and antioxidant (CAT, SOD, GSH, GSSG, GR, GPx) markers were determined.

**Results:**

Both CHOL-Si and CHOL-HxT diets enhanced the liver antioxidant status, reduced hepatosomatic index and increased SOD actvity. Hydrogen peroxide removal seemed to be involved, explaining that the value of redox index was even lower than C without changing the CAT activity. CHOL-Si results were quite better than CHOL-HxT in most measured parameters.

**Conclusions:**

Our study suggests that Si incorporated into RP matrix was able to counterbalance, more efficiently than HxT, the deleterious effect of consuming a high-saturated/high-cholesterol diet, by improving the liver antioxidant defenses in the context of NASH.

## Introduction

Pork products are an essential component of the diet in developed countries, its importance being associated with protein, vitamin and mineral intake in a balanced diet. However, pork consumption has been linked with major degenerative diseases (e.g. colon cancer and cardiovascular diseases), mainly related to saturated fatty acids and cholesterol levels [1]. Furthermore, the red meat consumption has been related to an increase in non-alcoholic steatohepatitis (NASH) [2, 3], the progressive form of non-alcoholic fatty liver disease (NAFLD) characterized, in addition to the steatosis, by inflammation and oxidative stress [4]. In order to achieve healthier meat and meat products, it is necessary to avoid undesired substances, reduce them to appropriate limits or increase (naturally or by programmed additions) other substances with proven beneficial properties (functional ingredients) [5, 6]. Different components exhibiting antioxidant activity, most isolated from plants (fruit, vegetables, seeds, spices, etc.), have been used as functional ingredients in meat based functional foods [6, 7]. Previous studies have shown the beneficial effects that consumption of restructured pork (RP) containing seaweed or glucomannan has on plasma cholesterol levels and endogenous antioxidant enzyme activities [8]. In this connection we propose the use of functional ingredients that, when incorporated into RP, may decrease cholesterol levels and oxidation and can be used to introduce elements to help prevent cardiovascular and neurodegenerative diseases.

Silicon (Si) is an important element that exists in nature in the form of oxides or silicates, is the second most available element on Earth and is easily incorporated into the human diet through a variety of agricultural products or drinking water (soluble as silicic acid, Si(OH)_4_). Apart from its physiological role in bone and cartilage formation and as an essential element for the brain, dietary Si affords protection against aluminum accumulation and consecutive oxidative damage in aged rat brains. This effect has been associated with protection against Alzheimer’s disease [9]. Silicic acid has also been found to induce down-regulation of endogenous antioxidant enzymes associated with aluminum administration [10]. Previous studies have proved that Si protects human SH-SY5Y cells from oxidative stress mediated by H_2_O_2_ [11, 12]. Studies have also demonstrated that Si helps prevent cardiovascular disease and acts as an immune system enhancer [13]. Si consumption is also inversely related with the onset of atherosclerosis [14]. The preventive effect of this mineral on atheroma formation is related to protection of arterial wall integrity and regulation of cholesterol levels, probably associated with the cholesterolemic effects of dietary fiber and its metabolism [15, 16]. Very recently our group has proven that Si added to RP strongly counterbalanced the negative effect of high-cholesterol-ingestion, functioning as an active hypocholesterolemic dietary ingredient in aged rats. In addition it helped to normalize VLDL composition and reduce VLDL oxidation in aged rats [17].

As a powerful antioxidant with proven functional properties, hydroxytyrosol (HxT) can be used as a potential functional food ingredient, especially in PUFA-enriched muscle foods. HxT capacity to inhibit lipid oxidation has been demonstrated in foodstuffs rich in fish lipids [18] and in pre-cooked beef and frankfurters enriched with *n*-3 polyunsaturated fatty acids [19, 20]. Also its *in vivo* antioxidant properties, by regulating arylesterase activity and oxidized LDL levels, have been proved on volunteers [21].

The hypothesis of the present work is that Si-enriched RP may act as a functional food by reducing liver oxidative status in aged rats fed a high-saturated/high-cholesterol diet as a model of NASH. These results would be joined to previous ones obtained *in vitro* [11, 12] and *in vivo* [17] opening discussion on the potential pleiotropic antioxidant effects of Si.

Thus, the aim of this research was to determine whether Si-RP exhibits antioxidant effects in comparison with HxT-RP in aged rats fed high-saturated/high-cholesterol diets.

## Experimental methods

### Diet preparation and experimental design

All experiments were performed in compliance with Directive 86/609/EEC of 24 November 1986 (amended by Directive 2003/65/EEC of 22 July 2003) on the protection of scientific research animals. This study was approved by the Spanish Science and Technology Advisory Committee (project AGL 2011-29644-C02-02) and by the Ethics Committee of the Universidad Complutense de Madrid (Spain). Twenty-four male Wistar rats were obtained from Harlan Laboratories Models, SL. (Barcelona, Spain). Animals were housed under controlled conditions (22.3 ± 1.8°C and 12 h light–dark cycle). Rats were fed commercial pellets (Panlab, Barcelona, Spain) during adaptation to environmental conditions and later distributed into four groups of six animals each. When rats were one year old and weighed approximately 500 g, they were housed individually and fed the experimental diets daily during eight weeks. Tap water, with 1.2 μg Si/mL, was provided *ad libitum* over the experimental period with an average consumption of 10 ml/100 g body weight/day. At the end of the experiment, rats were taken one at a time from each of the four groups, anesthetized with isoflurane (5%) and euthanized by extracting blood by heart puncture. Then, plasma was separated by centrifugation at 2000*g* for 10 min. Food consumption was measured daily and body weight once per week. Livers were collected and weighted immediately after blood extraction. Some samples were removed for histology, whereas others were preserved at -80°C until analysis. Feces were collected and weighed during the last week. No unexpected deaths of animals were registered during this study. Experimental diets were designed to have high-saturated/high-cholesterol/high-energy content, based on high amounts of sucrose and saturated fat. In addition, three of these diets were enriched with hypercholesterolemic inductors. Silicon (Si, Colloidal silicon dioxide, 113126, Merck Millipore, Darmstadt, Germany) and hydroxytyrosol (HxT, 2-(3,4-dihydroxyphenyl)-ethanol, SeproxBiotech, Madrid, Spain) were used in the preparation of diets. Minced lean pork and lard were purchased at a local store. Lean pork (849 g) and fat (151 g) were homogenized and RP was prepared following standard procedure [22] to obtain a similar composition to the one used in commercial sausages. For Si- and HxT-enriched RP preparations, components (1.3 and 3.6 g/kg fresh matter respectively) were previously mixed with lard to prevent subsequent dispersion. Si concentration of the RP was calculated according to Gozález-Muñoz et al.[10], that of HxT was based in previous studies [23, 24]. The different RP mixtures were freeze-dried in a LyoAlfa 10 freeze dryer (Telstar, Terrassa, Spain) [25] (S1 Table). Four experimental semi-synthetic diets were prepared: 1. Control RP diet (C), with no added cholesterol; 2. Cholesterol-enriched high-saturated/high-cholesterol control RP diet (CHOL-C), where maize starch was replaced by 1.26% cholesterol and 0.25% cholic acid; 3 and 4. Si- and HxT-RP cholesterol-enriched high-saturated/high-cholesterol diets (CHOL-Si and CHOL-HxT, respectively), similar to CHOL-C but with the enriched RP. To formulate experimental diets, 217 g of RP and 783 g of a modified semi-synthetic diet formulation (Panlab S.L., Barcelona, Spain; reference U8959 version 180) were mixed by serial dilutions until fully homogeneous. Diets were calculated to cover nutritional requirements at the final concentration (Table 1).

**Table 1:** Composition of the experimental diets fed to aged rats.

Diet	C	CHOL-C	CHOL-HxT	CHOL-Si
Protein (%)	21.6	21.6	21.6	21.6
Fat (%)	16.9	16.6	16.6	16.6
Cholesterol (g/kg)	0.14	16.3	16.3	16.3
SFA/MUFA/PUFA	30.9/32.2/13.1	30.9/32.2/13.1	30.9/32.2/13.1	30.9/32.2/13.1
Energy content, (MJ/kg)^1^	18.6	18.3	18.3	18.3
Total silicon content (μg/g)	430.0	330.0	374.0	1626.0
*Ingredients (g/kg)*				
Cornstarch	226.1	211.0	211.0	211.0
Casein	125.6	125.6	125.6	125.6
Maltodextrine	86.0	86.0	86.0	86.0
Sucrose	217.8	217.8	217.8	217.8
Soybean oil	44.0	44.0	44.0	44.0
Cellulose	31.4	31.4	31.4	31.4
AIN-93MX mineral mix^2^	44.0	44.0	44.0	44.0
AIN-93VX vitamin mix^3^	6.3	6.3	6.3	6.3
L-Cystine	1.9	1.9	1.9	1.9
Cholesterol	0	12.6	12.6	12.6
Cholic acid	0	2.5	2.5	2.5
Restructured pork^4^	217.0	217.0	217.0	217.0

C, control RP diet; CHOL-C, Cholesterol enriched high-saturated/high-cholesterol control RP diet; CHOL-HxT, hydroxytyrosol RP Cholesterol enriched high-saturated/high-cholesterol diet; Si, silicon RP Cholesterol enriched high-saturated/high-cholesterol diet.

^1^Data calculated considering as energy equivalent for carbohydrates: 16.73 kJ/g (4.0 kcal/g); fat, 37.65 kJ/g (9.0 kcal/g); protein 16.73 kJ/g (4.0 kcal/g).

^2,3^As reported by Reeves et al. [25].

^4^Restructured pork composition available as supporting information (S1 Table).

### Plasma cholesterol

Total plasma cholesterol was determined by the enzymatic colorimetric method using Spinreact kit (Sant Esteve de Bas, Girona, Spain).

### Silicon content, bioavailability, net absorption and digestibility

Si contents of the rat diets, liver, feces and drinking tap water were determined. Samples were digested in 70% nitric acid at 180°C (10 min ramp to 180°C and then maintained at 180°C for 20 min) in the microwave digestion system. Upon cooling, the digested samples and blanks were diluted with ultra-pure water and analyzed by inductively coupled plasma optical emission spectrometry (ICP-OES) for total Si concentration. The Si digestibility in rats was calculated by the following equation: Digestibility = (Si intake–Si fecal excretion)/Si intake; where Si intake is the total amount of silicon ingested by rats considering amounts in diet and tap water, and Si fecal excretion is the Si amount in feces. Si net absorption was calculated as the difference between total Si intake minus Si fecal excretion.

### Liver histology

The right lateral liver lobe was histological dissected and fixed in 10% formaldehyde and paraffin embedded, and 4 μm-thick lobe sections prepared. Sections were deparaffinized and stained with hematoxylin and eosin and observed at 20x magnification.

### Biochemical assays

Homogeneous samples of liver tissue were used for the biochemical assays. Oxidized (GSSG) and reduced (GSH) glutathione levels in liver homogenates were determined by the Hissin & Hilf method [26]. Liver tissue was homogenized in phosphate-EDTA buffer (0.1M sodium phosphate and 0.005M EDTA, pH 8) at 100 mg/mL, with the addition of 10 μL/mL of perchloric acid. It was then centrifuged at 10,000 rpm for 10 min at 4°C. Concentrations were calculated using a standard GSH and GSSG curve. Fluorescence was determined in a LS50 Perkin–Elmer (Baconfield, UK) fluorimeter at 350 and 420nm (excitation and emission wavelengths, respectively). Results were expressed as μg glutathione/mg protein. The calculated redox index (RI) indicates the antioxidant status of tissue obtained as follows: RI = GSSG/(GSH + GSSG).

Superoxide dismutase (SOD) activity was measured using the Nitroblue Tetrazolium (NBT) method with slight modifications [27]. This method is based on superoxide radical (.O_2_^-^) production by auto-oxidation of hydroxylamine hydrochloride which, in the presence of NBT, is reduced to nitrite. The reaction between nitrite and EDTA forms colorimetric complexes that were measured at 560 nm. Catalase (CAT) activity was measured as described by Aebi [28] using hydrogen peroxide as substrate. The decomposition of H_2_O_2_ was followed directly by a decrease in absorbance at 260 nm.

Enzyme activity was standardized to liver homogenate protein concentrations determined according to Bradford [29]. Final enzyme activity results were expressed as IU/mg protein.

### Western blotting and antioxidant enzyme levels

Total liver protein lysates were obtained and separated in 10% sodium dodecyl sulfate-poly-acrylamide gel electrophoresis (SDS-PAGE). Gels were then blotted onto PVDF Amersham Hybond-P membranes (GE Health-care, Buckinghamshire, UK) and incubated with their corresponding antibodies (A2228, A9917, C0979 and S2147 from Sigma–Aldrich, St. Louis, Missouri, USA; Ab59546 from Abcam, Cambridge, UK; sc-2004, sc-2490 and sc-32886 from Santa Cruz Biotechnology, Dallas, Texas, USA). β-actin was used as loading control. Blots were developed by enhanced chemiluminescence using an Amersham ECL Plus Western Blotting Detection Reagent (GE Health-care, Buckinghamshire, UK) according to manufacturer’s instructions.

### Extraction and quantification of RNA by RT-PCR

RNA samples were isolated from 100 mg of liver using TRI-Reagent (Sigma–Aldrich, St. Louis, Missouri, USA) according to the manufacturer’s instructions and treated with DNase I RNase-free reagents (Thermo Fisher Scientific, Waltham, Massachusetts, USA) to remove any contamination with genomic DNA. The yield and quality of RNA was assessed by measuring absorbance at 260, 280 and 310 nm and by electrophoresis on agarose gels (1%). Total RNA of each sample (1 μg) was reverse-transcribed to first-strand complementary DNA (cDNA) using a Revert Aid H Minus cDNA synthesis kit (Thermo Fisher Scientific, Waltham, Massachusetts, USA).

Relative *CAT*, *Mn-SOD*, *CuZn-SOD*, *GR* and Nuclear factor erythroid 2-related factor 2 (*Nrf2*) mRNA levels were quantified with a LightCycler Real Time PCR Detection System (Roche diagnostics, Indianapolis, Indiana, USA), using SYBR Green (Biotools, Madrid, Spain) as the fluorescent binding dye. Detection was monitored by measuring the increase in fluorescence throughout the cycles.

All sample mRNA levels were standardized to their *β-actin* values and results expressed as fold changes of the threshold cycle (Ct) value relative to controls using the 2^-ΔΔCt^ method [30]. PCR parameters were as follows: preincubation at 95°C for 10 min followed by 45 cycles of denaturation at 95°C for 10 s with an annealing temperature of 60°C for each couple primer, extension 72°C for 15 s and cooling at 40°C for 30 s. Primer sequences were as indicated in Table 2.

**Table 2:** Primer sequences used in RT-PCR.

*CAT*	sense: 5′-ATCAGGGATGCCATGTTGTT-3′ and antisense: 5′-GGGTCCTTCAGGTGAGTTTG-3′
*Mn-SOD*	sense: 5′-ACTGAAGTTCAATGGCGGG-3′ and antisense: 5′-TCCAGCAACTCTCCTTTGGG-3′
*CuZn-SOD*	sense: 5′-CTTCGAGCAGAAGGCAAGCG-3′and antisense: 5′-GACATGGAACCCATGCTCGC-3′
*GR*	sense: 5′-GGTTGGACTATGACAACATCCC-3′ and antisense: 5′-GCTTCATCTTCAGTGAGCCC-3′
*Nrf2*	sense: 5′-TTGTAGATGACCATGAGTCGC-3′ and antisense: 5′-GAGCTATCGAGTGACTGAGCC-3′
*β-actin*	sense:5′-CGGTCAGGTCATCACTATCGG-3′ and antisense:5′-TCCATACCCAGGAAGGAAGGC-3′

CAT, catalase; SOD, superoxide dismutase; GR, glutathione reductase; Nrf2, nuclear factor erythroid 2-related factor 2.

### Statistical analyses

All experiments were performed by triplicate. Data were analyzed using the SPSS Statistics v22 software (IBM Corporation, Somers, NY, US). Results were expressed as means and standard deviations. One way ANOVA followed by Bonferroni tests were used to assess the effect of HxT and Si. Where variances were assumed to be unequal, the T2 of the Tamhane post hoc test was applied. Student *t*-test was used to analyze cholesterol effect. Pearson product-moment correlations were performed to assess linear dependence between variables. Results were accepted as significant when *p*<0.05.

## Results

### Feed intake, body and liver weight

Table 3 shows the feed intake per week, rats consumed ~17 g diet/day. No intake differences were observed between groups. CHOL-C significantly increased liver weight but decreased final body weight (*p*<0.05) when compared to C. As a result of these differences, the hepatosomatic index was significantly higher (*p*<0.05) in CHOL-C when compared to C rats. Liver weight (*p* = 0.004) and final body weight (*p*<0.012) were significantly different in CHOL-C, CHOL-Si and CHOL-HxT groups. Final body weight was significantly lower (*p*<0.05) in CHOL-Si vs. CHOL-HxT, but showed no differences when compared to the CHOL-C group. Liver weight and the hepatosomatic index of CHOL-HxT rats were significantly lower (*p*<0.05) than those of CHOL-C but with no significant difference with CHOL-Si.

**Table 3:** Liver weight, hepatosomatic and redox index, plasma cholesterol, glutathione levels and silicon content, net absorption and digestibility.

	C		CHOL-C		CHOL-HxT		CHOL-Si		*p*	ANOVA
	Mean	*SD*	Mean	*SD*	Mean	*SD*	Mean	*SD*		
Final body weight (g)	657.00	65.08	455.17^a^	52.16	534.83^b^	53.16	509.94^a^	59.79	0.003	0.012
Feed intake (g/week)	120.44	5.38	118.63	9.52	109.53	8.08	119.01	10.49	0.645	0.098
Fecal excretion (g/week)	5.39	0.17	8.29	0.46	8.55	0.79	9.37	1.33	<0.001	0.267
Total silicon intake (mg/week)	52.76	3.15	38.31^a^	3.94	41.05^a^	2.09	197.21^b^	21.19	<0.001	<0.001
Fecal silicon content (mg/week)	4.38	0.34	5.39^a^	0.19	5.76^a^	0.64	30.22^b^	3.18	<0.001	<0.001
Silicon digestibility^1^	0.92	0.01	0.86	0.02	0.86	0.01	0.85	0.02	<0.001	0.472
Silicon net absorption^2^	48.39	3.23	32.92^a^	4.00	35.29^a^	1.54	166.98^b^	21.20	<0.001	<0.001
Liver weight (g)	14.08	1.70	25.24^a^	1.94	19.54^b^	2.44	22.85^ab^	2.83	<0.001	0.004
Liver silicon content (μg/g)	9.25	0.25	6.03^a^	0.13	5.15^a^	0.13	9.28^b^	1.86	<0.001	0.001
Hepatosomatic index^3^	2.15	0.21	5.56^a^	0.44	3.52^b^	0.49	4.37^b^	0.53	<0.001	0.015
Total hydroxytyrosol intake (mg/week)	-		-		226.49	11.53	-		-	-
Plasma cholesterol (mmol/L)	2.51	0.33	3.28^a^	0.34	3.01^ab^	0.28	2.69^b^	0.23	0.003	0.009
GSH (μg/mg protein)	2.80	0.80	3.01	0.91	2.90	0.90	2.80	0.40	0.684	0.952
GSSG (μg/mg protein)	4.40	0.61	6.00^a^	0.80	4.10^b^	0.91	3.80^b^	0.29	0.003	<0.001
Redox index^4^	0.61	0.07	0.67^a^	0.09	0.58^ab^	0.04	0.57^b^	0.01	0.282	0.025

Mean values and standard deviations, *n* 6. C, control RP diet; CHOL-C, cholesterol enriched high saturated/high cholesterol control RP diet; CHOL-HxT, hydroxytyrosol RP cholesterol enriched high saturated/high cholesterol diet; Si, silicon RP cholesterol enriched high saturated/high cholesterol diet. GSH, reduced glutathione; GSSG, oxidized glutathione. *p*, statistical comparison between C and CHOL-C. ANOVA, statistical effect of ingredients in cholesterol-fed groups. Values with different letters (a, b) in the same row indicate significant differences (at least *p*<0.05) between CHOL-C, CHOL-HxT and CHOL-Si groups.

^1^ Si digestibility = (Si intake − Si fecal excretion)/Si intake

^2^ Si net absorption = Si intake − Si fecal excretion

^3^ Hepatosomatic index = 100*(liver weight/body weight)

^4^ Redox index = GSSG/(GSH + GSSG)

### Total plasma cholesterol

The effect that Si- or HxT-RP and cholesterol addition in high-saturated/high-cholesterol diets had on plasma cholesterol is shown in Table 3. Dietary cholesterol significantly increased plasma cholesterol levels in CHOL-C (*p =* 0.003) when compared to C rats. Significant differences were observed between CHOL-C, CHOL-HxT and CHOL-Si groups (*p* = 0.009). The CHOL-HxT diet did not lower plasma cholesterol whereas the CHOL-Si diet lowered it by 18% (*p*<0.05) compared with CHOL-C rats.

### Silicon content, bioavailability, net absorption and digestibility

Si intake was significantly different between the C and CHOL-C groups (*p*<0.001). CHOL-Si animals ingested significantly more Si than CHOL-C and CHOL-HxT (*p*<0.001). Fecal Si excretion was higher in CHOL-C than in C group (*p*<0.001). Non-significant differences were observed in Si fecal excretion between the cholesterol added groups. Si net absorption was significantly different between the C and CHOL-C groups (*p*<0.001). CHOL-Si animals had higher net absorption than CHOL-C and CHOL-HxT (*p*<0.001). Si digestibility was higher (*p*<0.001) in C than CHOL-C animals while non-significant differences were found between CHOL-C, CHOL-Si and CHOL-HxT groups (*p* = 0.472). Liver silicon content was significantly higher in C vs. CHOL-C (*p*<0.001), no significant differences were observed between CHOL-C and CHOL-HxT; CHOL-Si did increased Si amount to similar values of C with significant differences with its CHOL-C and CHOL-HxT counterparts (Table 3).

### Histological examination of liver tissue

**Fig 1:**
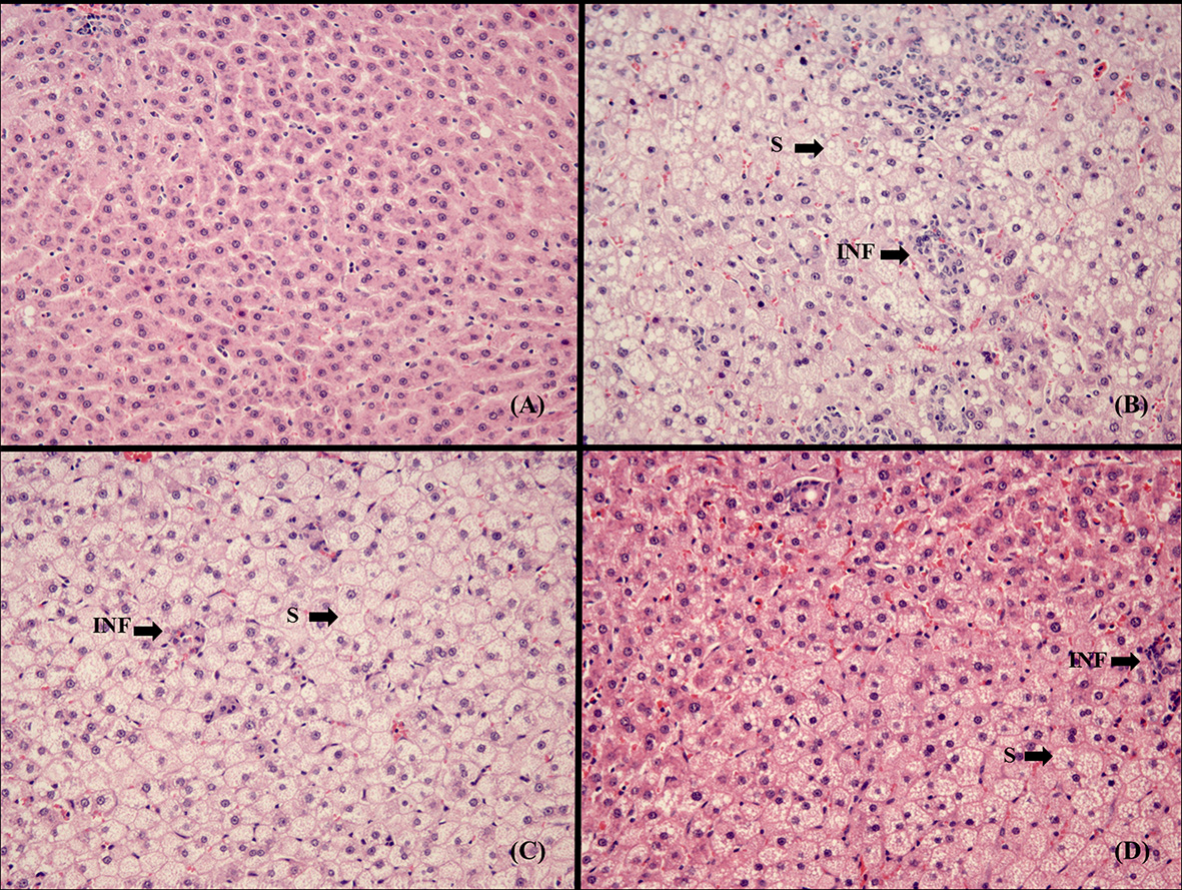
Histological evaluation of liver. Images represent liver sections at 20x magnification of the experimental groups. (A) C, control diet; (B) CHOL-C, cholesterol enriched high saturated/high cholesterol control diet; (C) CHOL-HxT, hydroxytyrosol RP cholesterol enriched high saturated/high cholesterol diet; (D) CHOL-Si, silicon RP cholesterol enriched high saturated/high cholesterol diet; S, hepatic steatosis; INF, inflammatory cell infiltration

Fig 1 summarized the most relevant findings in liver histology. C rats showed no evidence of hepatic steatosis (Fig 1A). In CHOL-C rats a large number of mixed-size vesicles were observed together with presence of mononuclear cell accumulation (Fig 1B). The incorporation of HxT or Si to the RP reduced the degree of steatosis and the presence of inflammatory cell infiltration, mainly in the CHOL-Si (Fig 1C and 1D respectively).

### Oxidized and reduced glutathione levels

CHOL-C rats exhibited higher GSSG levels (*p* = 0.003) than their C counterparts. However, no differences were observed in GSH levels (*p* = 0.684) or the redox index (*p* = 0.282) between these rats. CHOL-HxT and CHOL-Si rats had significantly lower GSSG values (at least *p*<0.05) than their CHOL-C counterparts. The redox index was significantly lower in CHOL-Si rats than CHOL-C ones (Table 3).

### Enzyme activities and expressions

Table 4 shows the effect of the experimental diets on antioxidant enzymes. CAT and SOD activity was significantly lower (*p*<0.001) in CHOL-C rats *vs*. C rats. *CAT*, *Mn-SOD*, *CuZn-SOD* and *Nrf2* values were lower (*p*≤0.001) in CHOL-C rats. Significant differences were found for activities and expressions between cholesterol-fed rat groups (ANOVA at least *p* = 0.044), except for GR (*p* = 0.513) expression and *CAT* (*p* = 0.663) activity. CHOL-Si exhibited the highest total SOD activity and *CuZn-SOD* expression (at least *p*<0.05) between all groups, and presented higher *Mn-SOD* and *Nrf2* expressions than CHOL-C rats (at least p<0.05). CHOL-HxT rats displayed higher SOD activity than their CHOL-C counterparts.

**Table 4:** Liver enzymes expressions and activities of aged rats fed cholesterol-enriched high saturated/high cholesterol diets containing hydroxytyrosol- or silicon-restructured pork.

	C		CHOL-C		CHOL-HxT		CHOL-Si		*p*	ANOVA
	Mean	*SD*	Mean	*SD*	Mean	*SD*	Mean	*SD*		
*CAT*										
Expression	1.00	0.09	0.75	0.12	0.41	0.31	0.37	0.31	<0.001	0.044
Activity (IU/mg protein)	12.32	0.89	4.81	1.48	5.25	1.84	4.28	2.08	<0.001	0.663
*SOD*										
*Mn-SOD* expression	1.00	0.11	0.48^a^	0.19	0.59^ab^	0.30	1.85^b^	1.04	0.001	0.020
*CuZn-SOD* expression	1.00	0.08	0.42^a^	0.10	0.81^a^	0.20	2.34^b^	1.51	<0.001	0.006
Activity (IU/mg protein)	5.71	0.79	3.20^a^	0.54	8.62^b^	2.17	13.06^c^	3.37	<0.001	<0.001
*GR*										
Expression	1.00	0.19	0.85	0.24	1.22	1.04	0.83	0.32	0.225	0.513
*Nrf2*										
Expression	1.00	0.21	0.18^a^	0.12	0.42^ab^	0.17	0.89^b^	0.64	<0.001	0.017

Mean values and standard deviations, *n* 6. C, control RP diet; CHOL-C, cholesterol enriched high saturated/high cholesterol control RP diet; CHOL-HxT, hydroxytyrosol RP cholesterol enriched high saturated/high cholesterol diet; Si, silicon RP cholesterol enriched high saturated/high cholesterol diet. CAT, catalase; SOD, superoxide dismutase; GR, glutathione reductase; Nrf2, nuclear factor erythroid 2-related factor 2. *p*, statistical comparison between C and CHOL-C diets. ANOVA, statistical effect of ingredient in cholesterol-fed groups. Values with different letters (a, b) in the same row indicate significant differences (at least *p*<0.05) between CHOL-C, CHOL-HxT and CHOL-Si groups.

### Enzyme Western blot levels

**Fig 2:**
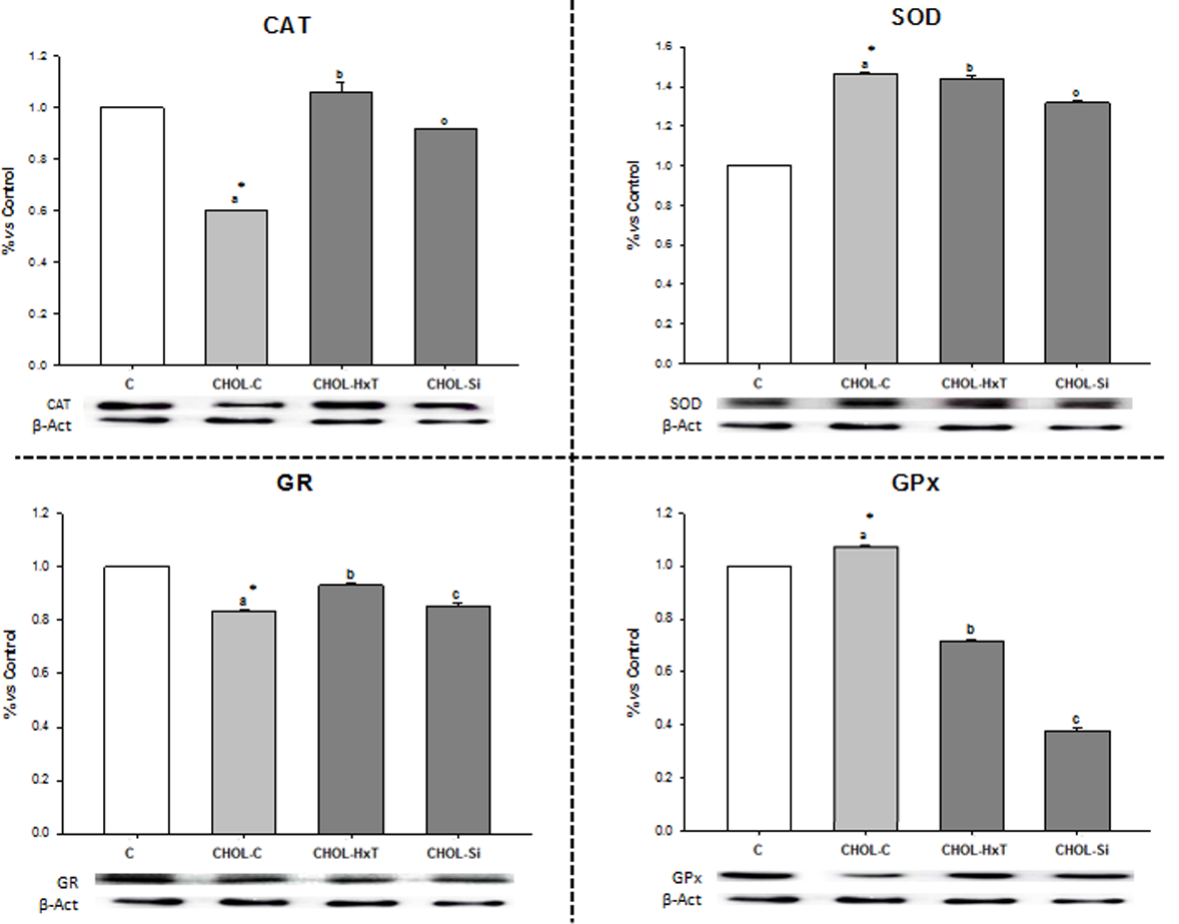
Effect of silicon or hydroxytyrosol-restructured pork on liver enzymatic antioxidants in aged rats fed high-saturated/high-cholesterol diets. Mean values and standard deviations (n 6). C, control diet; CHOL-C, cholesterol enriched high-saturated/high-cholesterol control diet; CHOL-HxT, hydroxytyrosol RP cholesterol enriched high-saturated/high-cholesterol diet; Si, silicon RP cholesterol enriched high-saturated/high-cholesterol diet. Statistical comparison between C and CHOL-C diets, * indicate significant differences (at least p<0.05). Bars with different letters indicate significant differences (at least p<0.05) between CHOL-C, CHOL-HxT and CHOL-Si groups. CAT, catalase; SOD, superoxide dismutase; GR, glutathione reductase; GPx, glutathione peroxidase. Results were calculated as percentage relative to control.

Fig 2 represents protein levels from antioxidant enzymes determined by Western-blot. SOD and GPx were higher while CAT and GR were lower (at least *p*≤0.05) in CHOL-C *vs*. C rats. Significant differences between CHOL-C, CHOL-Si and CHOL-HxT (ANOVA *p*≤0.001) were found, with CHOL-HxT exhibiting the highest protein level for CAT and GR. CHOL-Si increased CAT and GR levels with respect to CHOL-C but decreased all enzyme protein levels *vs*. CHOL-HxT.

### Correlations between Si intake, net absorption and bioavailability and the antioxidant enzymes

SOD activity and Mn-SOD and CuZn-SOD expressions (*r*>0.50; p<0.01) were positively and significantly correlated, while GPx protein level was negatively and significantly correlated (*r>*-0.62; p<0.01) with Si intake, net absorption and liver amount. *Nrf2* expression was positively and significantly correlated with Si intake and net absorption (*r*>0.55; p<0.05), and with Si liver amount (*r =* 0.71; p<0.001).

## Discussion

This study explored, for the first time, the antioxidant effects of Si, incorporated to RP, on a NASH model in aged rats. Present findings have led us to hypothesize a possible antioxidant mechanism by which Si acts in response to dietary atherogenic inductors.

Dietary combination of high-saturated/high-cholesterol, cholesterol and cholate in rodents causes histological features characteristics of NASH [31]. These include steatosis, inflammation and oxidative stress [32]. The increase in liver weight and hepatosomatic index, together with the histological features observed, agree with the criteria of NASH in the CHOL-C rats. Differences in body weight were not related to feed intake. The decrease in the body weight shown in CHOL-C group is in agreement with Beynen et al. [33], and is also typical of NASH models induced by cholesterol/cholate/high-fat diets [31]. A lower dietary intake was observed than previous reported in growing rats fed seaweed-RP diets [22] but similar to Nesic et al. [34], were the decrease is partially related to the anorexic effect of aging [35]. Results can also be related to the high-fat, high-energy content from the experimental diets.

Similarities in the dietary Si content and feed intake between C, CHOL-C and CHOL-HxT groups explain the absence of significant differences in total Si intakes and fecal excretion. Nonetheless, Si intake and liver Si content were higher as expected in case of CHOL-Si than in their CHOL-C and CHOL-HxT counterparts. Although cholesterol feeding affects negatively the Si net absorption and liver Si content (CHOL-C and CHOL-HxT *vs*. C groups), dietary Si positively counterbalanced this negative effect as CHOL-Si group showed similar liver Si content than the C group and approximately 50% higher than those found in CHOL-C and CHOL-HxT groups. Thus, results obtained appear, at least partially, related to the higher amount of Si net absorbed and available in liver.

Otherwise, CHOL-Si diet reduced the hypercholesterolemic effect of dietary cholesterol by bringing plasma levels down to similar values as the C diet. Si has been described as an anti-atheromatous agent thanks to a mechanism inhibiting lipid accumulation on vascular endothelium tissue. It therefore has an effect on total plasma cholesterol but not on liver lipid synthesis [15]. HxT exhibits biological antioxidant and anti-atherogenic properties [21, 36]. The results of this work show HxT’s ability to significantly reduce liver weight in comparison to the other cholesterol-enriched diets leading to a reduction in hepatic steatosis; as seen in Fig 1, the size, form and structure of hepatocyte vacuoles are much less pronounced. In fact, the usefulness of antioxidants to enhance the progression of the NASH has been proposed since oxidative stress is clearly involved in this disorder [37]. HxT also tended to reduce cholesterolemia (8%) although it was not significantly. Fki et al. [36] observed a similar tendency for cholesterolemia in rats fed cholesterol-enriched diets and HxT.

The age-related oxidative stress hypothesis suggests differences in the contribution of elements from the main antioxidant endogenous system in the case of older animals. In addition, dietary components may directly affect defense mechanisms protecting against exogenous stress leading to unexpected levels of non-enzymatic antioxidants (e.g. glutathione levels) and modifications of the enzymatic pathway. Studies have shown that GSH decreases with age whereas the GSSG and/or GSSG/GSH ratio increases [38]. Other studies have proved that elevated levels of dietary fat lead to high lipid peroxidation indirectly modifying GSH levels and glutathione enzyme activity [8, 39]. In fact, our findings showed that GSH levels were significantly lower than those of GSSG. It has been proved that dietary fat and hypercholesterolemia affect cell membrane integrity thus affecting defense against oxidative damage [40].

Taking into account just the control groups (C and CHOL-C) some correlations between the parameters tested helped us to clarify the results. There was a significant positive correlation between total SOD *vs*. CAT activities (*r* = 0.910; *p*<0.01) which could mean that hydrogen peroxide produced by SOD was removed mainly by CAT; in fact, a negative correlation between the CAT activity and the GSSG levels was found (r = -0.715; *p*<0.01). These results confirm the lower effectiveness of redox system related to the age [38].

**Fig 3:**
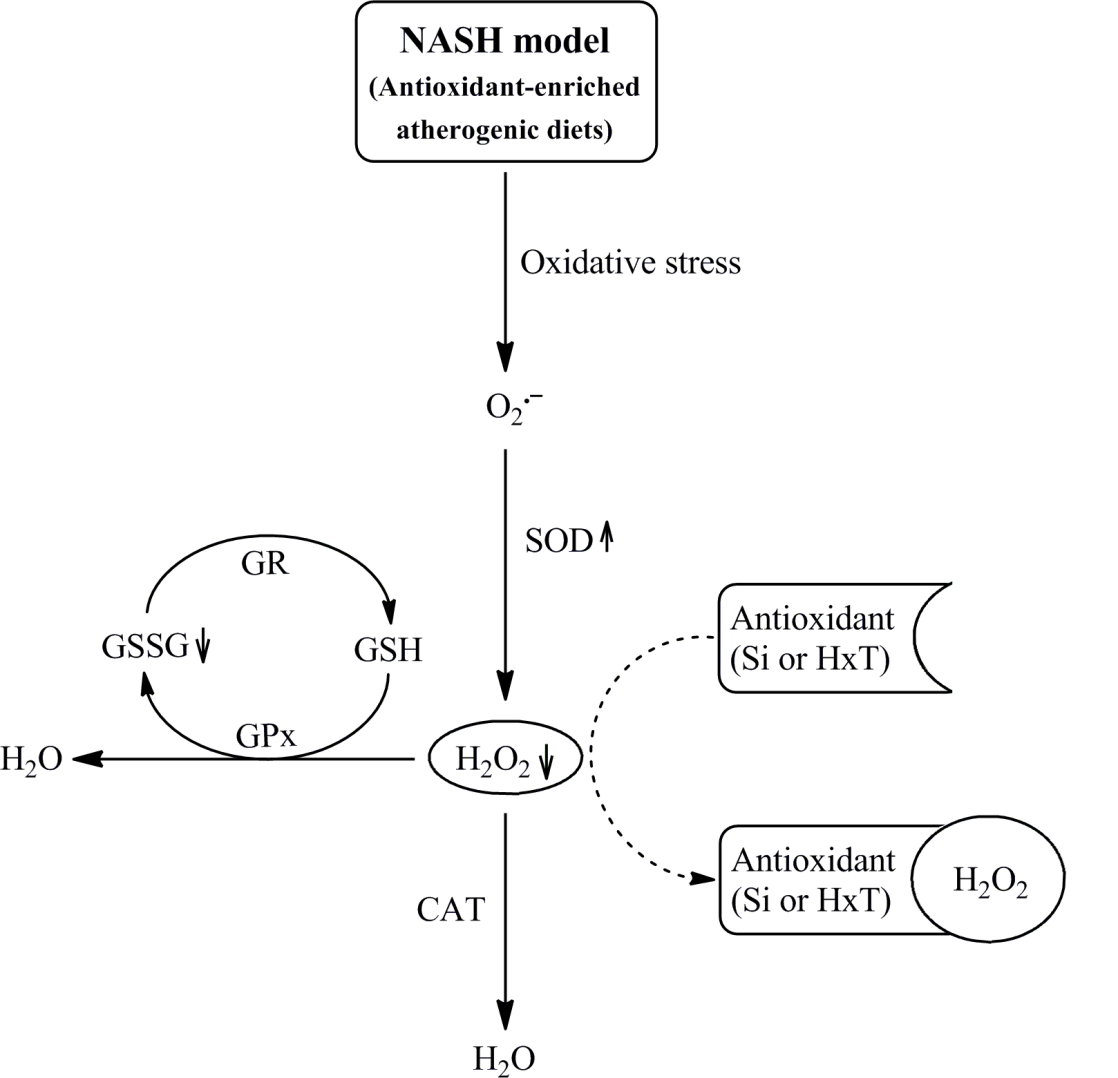
Proposed mechanism by which silicon (Si) or hydroxytyrosol (HxT) neutralize oxidative damage of cholesterol feeding. After high-saturated/high-cholesterol RP-diets consumption, oxidative stress is produced and SOD is activated. The formed hydrogen peroxide is then removed from the main antioxidant defense system by Si or HxT action, being in consequence not needed the overexpression of CAT or GPx pathways.

On the other hand, CHOL-C rats showed lower total SOD and CAT enzymatic activities than C rats in similar proportion as reported in previous publications in growing rats [22]. Furthermore, the redox index increased due to a significantly reduction in GR levels and expression, suggesting that cholesterol feeding affects more the redox index in 1 year old rats than in younger ones [41]. The incorporation of Si or HxT to RP reduced oxidative damage in this NASH model. The CHOL-Si diet induced the highest total SOD activity and Mn-SOD and CuZn-SOD expression, but the lowest SOD protein levels. These results could be explained, at least partially, because an increase of enzyme activity is sufficient to reduce oxidative damage and would make the synthesis of more protein unnecessary. The increased total SOD activity would lead to an increase in hydrogen peroxide levels that was not paralleled to those of CAT or GPx. In line with this asseveration, Si intake, Si net absorption and liver Si content were positive correlated with total SOD activity, Mn-SOD and CuZn-SOD expressions but negatively with GPx protein level. Thus, other alternative mechanism can be needed to remove the overproduced hydrogen peroxide. Because the redox index was kept at C levels in CHOL-Si rats and a significant negative correlation between total SOD activity *vs*. GSSG (*r* = 0.956; *p*<0.01) was found, it can be suggested that dietary and liver Si neutralized the oxidative damage of cholesterol feeding by removing the hydrogen peroxide, being in consequence not needed the overexpression of CAT or GPx pathways (Fig 3). Similar results were observed by Vidé et al. [42] who found that SOD and GPx activities were enhanced after supplementations with Si, in the frame of a high-fat diet. However this induction did not prevent hepatic ROS accumulation, suggesting that GPx activation was not the main mechanism to prevent oxidative damage and therefore an alternative mechanism should be considered. These differences could also be due to the composition of the diet which cause different liver damage degree, but also due to the differences on Si content, that is much higher in our experiments. Our hypothesis is based on results of previous studies [11, 12] performed in cell culture where the ability of Si to capture hydrogen peroxide was demonstrated.

Regarding to CHOL-HxT rats, SOD was significantly increased with respect to CHOL-C ones, although the increase did not reach that of CHOL-Si rats. SOD increase was not paralleled to those of CAT or GPx suggesting that HxT could exert similar effect of Si in relation to hydrogen peroxide neutralization. The ability of HxT to remove hydrogen peroxide was previously proposed by Manna et al. [43]. Likewise, CAT levels were significantly higher respect to CHOL-Si rats, but the activity was not modified. Moreover, despite CHOL-HxT animals showed significantly higher GR and GPx levels than their CHOL-Si counterparts; the redox index was not changed Fig 3.

Nrf2 is a key component in cellular redox homeostasis involved in the attenuation of oxidative stress-related pathological processes. Under normal conditions, activation of such processes is related to GSH synthesis, lipid metabolism and inflammation inhibition [44, 45]. Previous studies have emphasized the role of Nrf2 in NASH progression, as the knockout mice fed HF and high-cholesterol diet showed impaired steatohepatitis [46]. Moreover, it has been reported that Nrf2 levels decrease with age [47] and a microarray study by Yates et al. [48] showed that the genetic or pharmacological activation of Nrf2 represses the expression of key enzymes involved in fatty acid synthesis, with concomitant reduction in the levels of hepatic lipids. Nrf2 appears to protect the liver against steatosis by inhibiting lipogenesis and promoting fatty acid oxidation [49]. Our findings suggest that Si and HxT are exogenous stimuli that promote the activation of Nrf2 as an oxidative response to high-cholesterol/high fat diets and aging damage. No significant differences were observed between CHOL-Si and CHOL-HxT, although CHOL-Si did increase the expression of *Nrf2* similar to the control. Significant positive correlations between *Nrf2 vs*. SOD (*r* = 0.434; *p*<0.05) and *Nrf2 vs*. CAT (*r* = 0.430; *p*<0.05) were observed in our experimental conditions. Previous studies have demonstrated that HxT offers protection against the decrease in total protein level expression of Nrf2 caused by acrolein [50]. Incorporation of Si- or HxT-RP to the diet may activate Nrf2 and spark the expression of phase II detoxifying enzymes conferring additional antioxidant protection, thus, enhancing the antioxidant defense system and damage prevention of reactive oxygen species. In fact, correlations found between *Nrf2* and Si intake, Si net absorption and liver Si content support the protective role of Si against liver steatosis [49] and oxidative stress [44, 45].

This paper has the merit of being the first to study the effect that Si incorporated into RP has on a number of antioxidant markers in aged rats fed high saturated/high cholesterol diets. Moreover, the study was long enough to assess quite permanent effects. Si was tested against a recognized antioxidant ingredient. As no Si daily allowance recommendations are available yet, this paper opens the discussion on the addition of Si to meat products to increase health effects related to CVD diseases. We would note, however, that only one Si concentration was tested. We would suggest further study of the effect of Si on elderly humans consuming unhealthy diets.

In conclusion, when incorporated into pork matrices in the context of high saturated/high cholesterol diets, Si has the ability to act as a protector of oxidative damage in aged rats. This protection was more effective than HxT and suggests an inhibition effect over ROS (mainly hydrogen peroxide) and regulation of antioxidant enzymes expressions and activities giving rise to lower GSSG levels. These results, together with previous one of our group [11, 12, 17], stress the importance of incorporating Si into the diet; nonetheless, further studies on other animal and human models should be performed.

## Supporting Information

S1 Table Composition of the Restructured Pork (RP) incorporated to the experimental diets fed 1-year old male Wistar rats. C, control RP diet; CHOL-C, cholesterol enriched high saturated/high cholesterol control RP diet; CHOL-HxT, hydroxytyrosol RP cholesterol enriched high saturated/high cholesterol diet; CHOL-Si, silicon RP cholesterol enriched high saturated/high cholesterol diet.
(DOCX)
