# Supplementary material for: Effects of Silicon vs. Hydroxytyrosol-Enriched Restructured Pork on Liver Oxidation Status of Aged Rats Fed High-Saturated/High-Cholesterol Diets
Source: PLoS One. 2016 Jan 25;11(1):e0147469. doi: 10.1371/journal.pone.0147469 (PMC4726576; doi:10.1371/journal.pone.0147469)
Supplement: S1 Table — C, control RP diet; CHOL-C, cholesterol enriched high saturated/high cholesterol control RP diet; CHOL-HxT, hydroxytyrosol RP cholesterol enriched high saturated/high cholesterol diet; CHOL-Si, silicon RP cholesterol enriched high saturated/high cholesterol diet. (DOCX) [file pone.0147469.s001.docx]

**Supporting information**

**S1 Table. Composition of the Restructured Pork (RP) incorporated to the experimental diets fed 1-year old male Wistar rats.** C, control RP diet; CHOL-C, cholesterol enriched high saturated/high cholesterol control RP diet; CHOL-HxT, hydroxytyrosol RP cholesterol enriched high saturated/high cholesterol diet; CHOL-Si, silicon RP cholesterol enriched high saturated/high cholesterol diet.

| **Restructured pork components** | **C and CHOL-C** | | | **CHOL-HxT** | | **CHOL-Si** | |
| --- | --- | --- | --- | --- | --- | --- | --- |
| Protein (*%*) | | 15.6 | 15.6 | | 15.6 | |  |
| Fat (*%*) | | 21.9 | 21.9 | | 21.9 | |  |
| Water (*%*) | | 62.5 | 62.5 | | 62.5 | |  |
| Cholesterol (*g/kg*) | | 0.65 | 0.65 | | 0.65 | |  |
| SFA/MUFA/PUFA ratio | | 38.7/41.9/9.7 | 38.7/41.9/9.7 | | 38.7/41.9/9.7 | |  |
| Ingredients (*g/kg*) | |  |  | |  | |  |
| Lean pork | | 801.3 | 799.5 | | 800.7 | |  |
| Lard | | 152.2 | 152.2 | | 152.2 | |  |
| NaCl | | 5.1 | 5.1 | | 5.1 | |  |
| Sodium nitrite | | 1.2 | 1.2 | | 1.2 | |  |
| STP | | 0.1 | 0.1 | | 0.1 | |  |
| Silicon dioxide | | 0.0 | 0.0 | | 1.3 | |  |
| Hydroxytyrosol | | 0.0 | 3.6 | | 0.0 | |  |
| Water | | 40.1 | 38.3 | | 39.4 | |  |

C, control RP diet; CHOL-C, cholesterol enriched high saturated/high cholesterol control RP diet; CHOL-HxT, hydroxytyrosol RP cholesterol enriched high saturated/high cholesterol diet; CHOL-Si, silicon RP cholesterol enriched high saturated/high cholesterol diet.
